# Macrophages and Dendritic Cells Emerge in the Liver during Intestinal Inflammation and Predispose the Liver to Inflammation

**DOI:** 10.1371/journal.pone.0084619

**Published:** 2014-01-02

**Authors:** Yohei Mikami, Shinta Mizuno, Nobuhiro Nakamoto, Atsushi Hayashi, Tomohisa Sujino, Toshiro Sato, Nobuhiko Kamada, Katsuyoshi Matsuoka, Tadakazu Hisamatsu, Hirotoshi Ebinuma, Toshifumi Hibi, Akihiko Yoshimura, Takanori Kanai

**Affiliations:** 1 Division of Gastroenterology and Hepatology, Department of Internal Medicine, Keio University School of Medicine, Tokyo, Japan; 2 Department of Microbiology and Immunology, Keio University School of Medicine, Tokyo, Japan; 3 Research Laboratory, Miyarisan Pharmaceutical, Tokyo, Japan; 4 Department of Pathology and Comprehensive Cancer Center, University of Michigan Medical School, Ann Arbor, Michigan, United States of America; University of Chicago, United States of America

## Abstract

The liver is a physiological site of immune tolerance, the breakdown of which induces immunity. Liver antigen-presenting cells may be involved in both immune tolerance and activation. Although inflammatory diseases of the liver are frequently associated with inflammatory bowel diseases, the underlying immunological mechanisms remain to be elucidated. Here we report two murine models of inflammatory bowel disease: RAG-2^−/−^ mice adoptively transferred with CD4^+^CD45RB^high^ T cells; and IL-10^−/−^ mice, accompanied by the infiltration of mononuclear cells in the liver. Notably, CD11b^−^CD11c^low^PDCA-1^+^ plasmacytoid dendritic cells (DCs) abundantly residing in the liver of normal wild-type mice disappeared in colitic CD4^+^CD45RB^high^ T cell-transferred RAG-2^−/−^ mice and IL-10^−/−^ mice in parallel with the emergence of macrophages (Mφs) and conventional DCs (cDCs). Furthermore, liver Mφ/cDCs emerging during intestinal inflammation not only promote the proliferation of naïve CD4^+^ T cells, but also instruct them to differentiate into IFN-γ-producing Th1 cells *in vitro*. The emergence of pathological Mφ/cDCs in the liver also occurred in a model of acute dextran sulfate sodium (DSS)-induced colitis under specific pathogen-free conditions, but was canceled in germ-free conditions. Last, the Mφ/cDCs that emerged in acute DSS colitis significantly exacerbated Fas-mediated hepatitis. Collectively, intestinal inflammation skews the composition of antigen-presenting cells in the liver through signaling from commensal bacteria and predisposes the liver to inflammation.

## Introduction

Patients with inflammatory bowel diseases (IBD) are susceptible to developing extraintestinal disorders in the joints, eyes, skin, or liver [1]. For example, primary sclerosing cholangitis (PSC) has been diagnosed in 3.7% of patients with ulcerative colitis [2] and in 3.4% of those with Crohn’s disease [3]. The liver and the biliary system are the usual sites for extraintestinal lesions, despite being located between systemic and portal circulations. The portal vein contains a large amount of gut-derived products, such as short-chain fatty acids and microbe-associated molecular patterns (MAMPs) [4]. Although MAMPs, such as LPS from gram-negative commensal bacteria, act as a strong stimulants for antigen-presenting cells (APCs) [5], the liver has been shown to be an immunologically tolerant organ [6], [7]. The portal venous tolerance system is regulated by various immune compartments which contain natural killer (NK) cell, natural killer T (NKT) cell, and regulatory T cells, macrophages (Mφ) such as Kupffer cells, and dendritic cells (DCs) [8]. Recent studies have shown that plasmacytoid DCs (pDCs), a subgroup of resident DCs, induce anergy or rapid depletion of antigen-specific T cells in the liver via a CD4^+^ T cell-independent mechanism [9], [10]. These findings suggest that regulation and dysregulation of APCs in the liver contribute to liver tolerance and inflammation, respectively. However, the mechanisms of immune regulation and dysregulation in human IBD and experimental colitis models are not yet fully understood. A few studies have focused on the role of gut microbiota and MAMPs in promoting high-fat induced steatohepatitis [11], however, mechanism of immunological dysregulation in the liver during colitis still remains to be elucidated.

Our group has previously reported that increased numbers of Mφs and conventional DCs (cDCs) in experimental colitis models [12] and human IBD [13] have pro-inflammatory characteristics through excess production of IL-12 and IL-23 in response to bacteria. This leads to the development of Th1 immunity in inflamed intestinal mucosa. More recently, we demonstrated that migrating macrophages contribute to the induction of acute liver inflammation in murine hepatitis models [14].

To clarify hepatic immunological regulation under colitic conditions, we used three murine IBD models: (1) RAG-2^−/−^ mice adoptively transferred with splenic CD4^+^CD45RB^high^ T cells from wild-type (WT) mice [15]; (2) an acute dextran sulfate sodium (DSS)-induced colitis model [16]; and (3) IL-10^−/−^ mice [17] that spontaneously develop chronic IBD-like colitis.

## Materials and Methods

### Mice

WT C57BL/6J mice (8–12 weeks old) were purchased from Japan Clea (Tokyo, Japan). C57BL/6-Ly5.1 mice and RAG-2^−/−^ mice were obtained from Taconic Laboratory (Hudson, NY, USA) and the Central Laboratories for Experimental Animals (Kawasaki, Japan), respectively. IL-10^−/−^ mice were purchased from Jackson Laboratories (Bar Harbor, Maine, USA). Recipient RAG-2^−/−^ mice were used when they were 6 or 14 weeks old. Colitic IL-10^−/−^ mice were used when they were 20 weeks old. Germ-free (GF) C57BL/6-Ly5.2 mice (8 weeks old) were purchased from Sankyo Laboratories (Tokyo, Japan). GF mice were maintained in vinyl isolators within the gnotobiotic facility of the Miyarisan pharmaceutical company (Tokyo, Japan).

All experiments were approved by the Committee on the Ethics of Animal Experiments of Keio University School of Medicine, and conducted in accordance with institutional guidelines and Home Office regulations. [No. 24-026-1].

### Adoptive Transfer Studies

For adoptive transfer, CD4^+^ T cells were isolated from spleen cells of C57BL/6-Ly5.1 mice using the anti-CD4 (L3T4)-MACS system (Miltenyi Biotec, Auburn, CA, USA) according to the manufacturer’s instructions. Enriched CD4^+^ T cells (96–97% pure) were labeled with PE-conjugated anti-mouse CD4 (RM4-5; BD bioscience, San Diego, CA, USA) and FITC-conjugated anti-CD45RB (16A; BD bioscience). CD4^+^CD45RB^high^ cells were purified (>98.0%) using a FACS Aria (Becton Dickinson Co.). RAG-2^−/−^ mice (6 weeks old) were injected i.p. with 3×10^5^ CD4^+^CD45RB^high^ T cells. At 6 weeks post-transfer, these mice developed a wasting disease and colitis as previously reported [15].

For adoptive retransfer, lamina propria (LP) CD4^+^ T cells were isolated from colon LP mononuclear cells of RAG-2^−/−^ RB^high^ mice using the anti-CD4 (L3T4)-MACS system. Isolated LP CD4^+^ T cells were injected i.p. into RAG-2^−/−^ mice (RAG-2^−/−^ LP CD4^+^ mice). Mice were maintained under specific pathogen-free (SPF) conditions in the Animal Care Facility of Keio University.

### DSS-induced Colitis Model

Mice were treated under SPF conditions with 2% DSS (MW 50 kDa; Ensuiko Sugar Refining Co., Yokohama, Japan) in drinking water for 7 days (>4 mice per group). Mice were treated under GF conditions with 1% DSS in drinking water for 7 days followed with regular drinking water for 3 days (>4 mice per group).

### Animal Models of Liver Injury

Concanavalin A (Con A, type IV) was purchased from Sigma-Aldrich (St. Louis, MO, USA). Intravenous injections of Con A (20 mg/kg) were administered into the tail vein of animals 10 h before examination. The Fas-activating antibody Jo2 (0.3 mg/kg of body weight; BD bioscience) was injected i.p. and mice were sacrificed 6 h later [18], [19].

### Preparation of Liver Mononuclear Cells

Liver mononuclear cells were separated from the liver as previously described [20]. Livers were perfused through the portal vein with PBS, then minced and passed through a 100 µm nylon mesh. The filtrate was centrifuged at 50×*g* for 1 min, and the supernatant washed once. Cells were suspended in Histopaque solution (Sigma-Aldrich) and overlaid on HBSS. After centrifugation (780×*g* for 20 min), cells were collected from the upper phase.

### Preparation of LP Mononuclear Cells

Cell isolation was performed as previously described [21]. Dissected colon mucosa was incubated with Ca^2+^, Mg^2+^-free HBSS containing 1 mM DTT (Sigma-Aldrich) and 5 µM EDTA (Gibco) for 30 min, then treated with 3 mg/ml collagenase (Roche Diagnostics GmbH, Germany) and 0.01% DNase (Worthington Biomedical Co., Freehold, NJ, USA) for 1 h. Cells were pelleted twice through a 40% isotonic Percoll solution and then subjected to Ficoll-Hypaque density gradient centrifugation (40%/75%).

### Histological Examination

Liver and colon were fixed in 10% formalin and embedded in paraffin. Sections were stained with H&E and then examined. Histological examination of acute colitis was performed as described previously [22]. Briefly, histological activity score was assessed as the sum of three parameters as follows: extent, 0–3 (0, none; 1, mucosa; 2, mucosa and submucosa; 3, transmural); inflammation, 0–3 (0, none; 1, slight; 2, moderate; 3, severe); crypt damage, 0–4 (0, none; 1, basal 1/3 lost; 2, basal 2/3 lost; 3, only surface epithelium intake; 4, entire crypt and epithelium lost). The score of each parameter was multiplied by a factor of 1–4 (1, 0–25%; 2, 26–50%; 3, 51–75%; 4, 76–100%) according to the percentage of epithelial involvement.

### Flow Cytometry

After blocking with anti-FcR (CD16/32, BD bioscience) for 20 min, cells were incubated with specific mAbs at 4°C for 30 min. The following mAbs were used: anti-mouse CD3e-APC-Cy7; anti-CD4-PE-Cy7; anti-NK1.1-APC; anti-CD11b-PE-Cy7; anti-CD11cFITC; 7-AAD; anti-PDCA-1-APC; anti-CCR9-PE; anti-IFN-γ-FITC; and anti-IL-17-APC (eBioscience, BD bioscience). Background fluorescence was assessed by staining with irrelevant anti-rat isotypes (BD bioscience). Stained cells were analyzed by flow cytometry (FACS Canto II, Becton Dickinson Co.) and data analyzed using FlowJo software (Tree Star Inc.) [12].

### Quantitative RT-PCR (qPCR)

All qPCR assays were performed as described previously [14]. RNA was extracted from LP mononuclear cells using TRIzol reagent (Invitrogen, Carlsbad, CA, USA) and cDNA was synthesized from 100 ng of total RNA using TaqMan® Reverse Transcription Reagents (Applied Biosystems, Foster City, CA, USA). Reverse transcription was performed at 25°C for 10 min, 48°C for 30 min, and then 95°C for 5 min. cDNA was analyzed by qPCR using TaqMan® Universal PCR Master Mix (Applied Biosystems) in an Applied Biosystems StepOne™/StepOnePlus™ Real-Time PCR System. Cycling conditions for PCR amplification were 50°C for 2 min and 95°C for 10 min, followed by 40 cycles of 95°C for 10 s, then 60°C for 1 min. Relative quantification was achieved by normalizing to the β-actin gene (Applied Biosystems). The following probes were purchased from Applied Biosystems: *Ifng* (99999071_m1), *Tnf* (99999068_m1) and *Actb* (01205647_g1).

### *In vitro* Proliferation Assays

APCs, PDCA-1^+^ pDCs from the livers of C57BL/6 mice, CD11b^+^ Mφs from the inflamed livers of Con A-injected C57BL/6 mice (Con A Mφs), IL-10^−/−^ mouse Mφs, and DSS-treated C57BL/6 mouse Mφs (DSS Mφs) were isolated using a FACS Aria (Becton Dickinson Co.). Enriched naïve CD4^+^ splenocytes obtained from OT-II mice were sorted using a CD4^+^ CD62L^+^ T Cell Isolation Kit II (Miltenyi Biotech, Auburn, CA, USA) and labeled with 1 mM CFSE (Molecular Probes, Eugene, OR, USA) for 10 min at 37°C, followed by the addition of 1.0 ml of FCS for 2 min and washed three times in PBS. CFSE-labeled CD4+ naïve cells (1×10^5^ cells/well) were co-cultured with pDCs or Mφs (2×10^4^ cells/well) in 96-well round-bottom plates for 72 h in the presence of OVA peptides (1 µM). After incubation, cells were collected, incubated with anti-CD4-PE-Cy7 and anti-CD3e-APC-Cy7 and analyzed by FACS; 7-AAD was added to exclude dead cells. Proliferation analysis is based on division times of CFSE^+^CD4^+^ T cells.

Unlabeled CD4^+^ naïve T cells (1×10^5^ cells/well) were also co-cultured with pDCs or Mφs (2×10^4^ cells/well) for 120 h in the presence of OVA peptides followed by incubation with anti-IFN-γ and/or anti-IL-17 mAbs, and then treated with a Cytofix/Cytoperm kit (BD bioscience). Culture supernatant was collected and analyzed with the BD™ Cytometric Beads Array Mouse Th1/Th2/Th17 Cytokine Kit (Becton Dickinson Co.).

### Statistical Analysis

Results are expressed as mean ± SEM. Data groups were analyzed with GraphPad Prism using Tukey-Kramer test and Student’s *t*-tests. A *P*-value less than 0.05 was considered statistically significant.

## Results

### Accumulation of Mononuclear Cells was Induced in the Liver of Mice with Chronic Colitis

**Figure 1:**
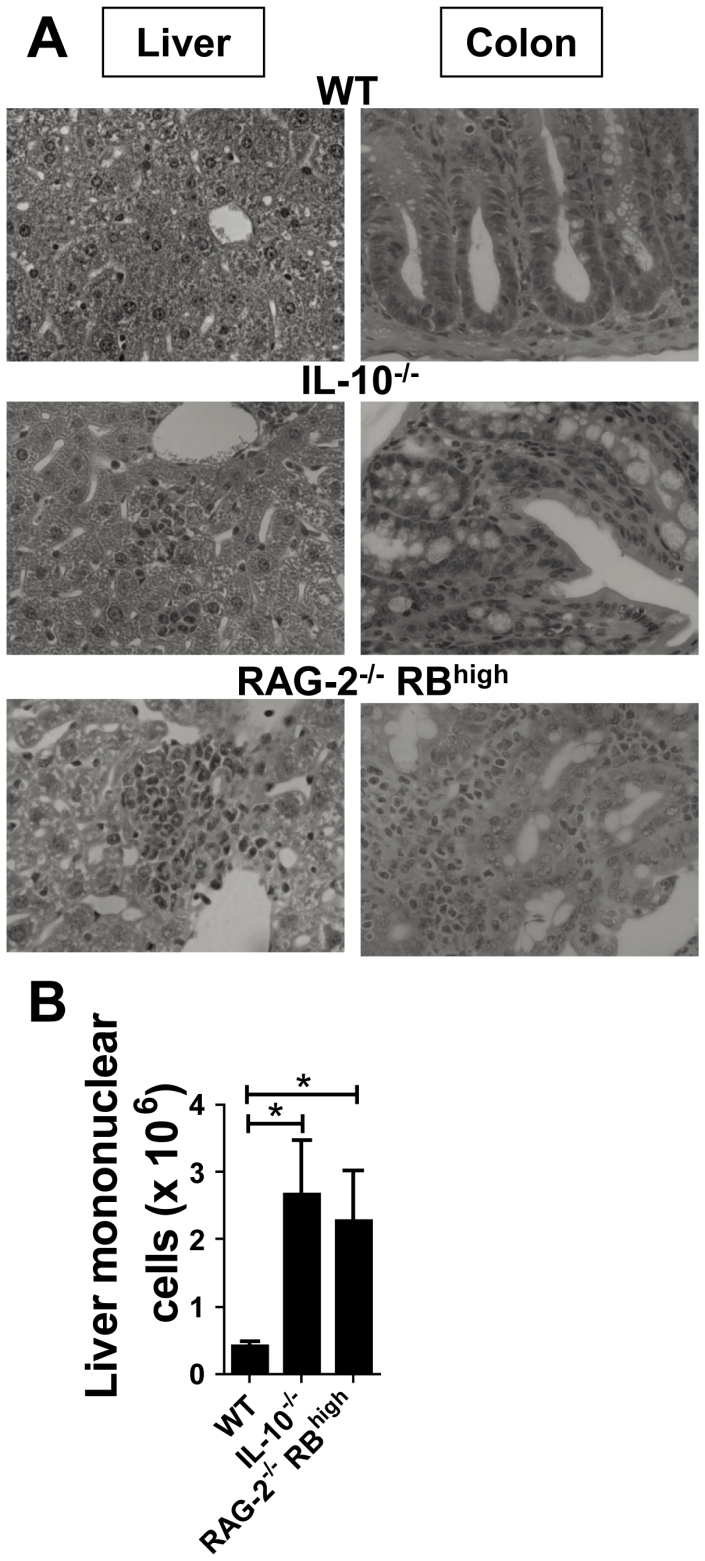
Accumulation of mononuclear cells in the liver develops in chronic colitis models. (**A**) H&E specimens of the liver (left) and colon (right) derived from WT, IL-10^−/−^ mice, and RAG-2^−/−^ RB^high^ mice. Magnification: ×100 (left) and ×400 (right). (**B**) Absolute number of hepatic mononuclear cells. FACS data are representative of three independent experiments. Values are expressed as means ± SEM for each group. WT (*n* = 5), IL-10^−/−^ mice (*n* = 7) and RAG-2^−/−^ RB^high^ mice (*n* = 4). *P<0.05.

To investigate hepatic immunological regulation in the colitic condition, we first used two murine IBD models, RAG-2^−/−^ mice adoptively transferred with splenic CD4^+^CD45RB^high^ T cells from WT mice (RAG-2^−/−^ RB^high^ mice) and IL-10^−/−^ mice. Consistent with previous reports [15], RAG-2^−/−^ RB^high^ mice showed severe colitis, and infiltration of mononuclear cells in the portal vein area of the liver (Fig. 1A). This was not observed in WT mice. IL-10^−/−^ mice spontaneously developed colitis, characterized by prominent epithelial hyperplasia with leukocyte infiltration into the liver (Fig. 1A). Consistently, the absolute number of liver mononuclear cells in both colitis models was significantly increased when compared with age-matched C57BL/6 mice (Fig. 1B). Liver enzymes (aspartate aminotransferase and alanine aminotransferase) demonstrated no significant changes between WT mice and the two colitis groups (data not shown).

### Chronic Colitis is Associated with APC Balance in the Liver

**Figure 2:**
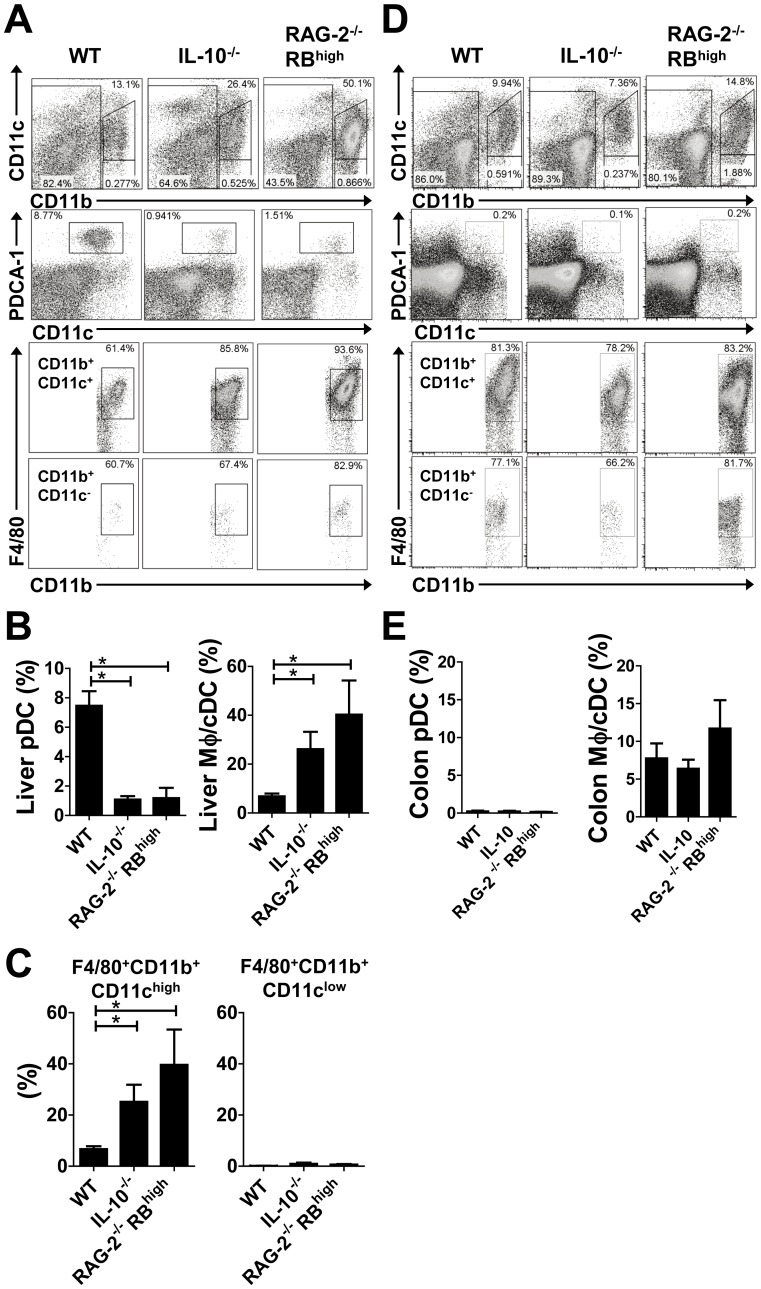
Chronic intestinal inflammation was associated with reciprocal changes in the balance of APCs. (**A**) Flow cytometry results related to mononuclear cells isolated from the livers of WT (left column), IL-10^−/−^ (middle), and RAG-2^−/−^ RB^high^ (right) mice. Dead cells were excluded with 7AAD staining, followed by proper use of a FSC/SSC gate. CD11b^−^, CD11b^+^CD11c^high^, and CD11b^+^CD11c^low^ cells were gated from the cells shown in the first row. CD11b^−^ cells are shown in the second row and PDCA-1^+^CD11b^−^CD11c^int^ cells were analyzed. The expression of F4/80 in CD11b^+^CD11c^high^ cells and CD11b^+^CD11c^low^ cells are analyzed in the third row and the fourth row. (**B**) Proportion of PDCA-1^+^CD11b^−^CD11c^int^ pDCs and F4/80^+^CD11b^+^ Mφ/cDCs among whole mononuclear cells. (**C**) Proportion of F4/80^+^ CD11b^+^CD11c^high^ Mφ/cDCs and F4/80^+^ CD11b^+^CD11c^low^ Mφ/cDCs among whole mononuclear cells. (**D**) Flow cytometry analysis of mononuclear cells isolated from the colons of WT (left column), IL-10^−/−^ (middle), and RAG-2^−/−^ RB^high^ (right) mice. (**E**) Proportion of PDCA-1^+^CD11b^−^CD11c^+^ pDCs and F4/80^+^CD11b^+^CD11c^−^ Mφ/cDCs among whole mononuclear cells. FACS data are representative of three independent experiments expressed as means ± SEM for each group. WT (*n* = 4), IL-10^−/−^ (*n* = 4) and RAG-2^−/−^ RB^high^ (*n* = 3) mice. *P<0.05.

Since it has been reported that Mφ/cDCs and pDCs represent subgroups of APCs differentiated from Mφ/DC precursors [23], we further investigated the composition of APCs in the liver. Analysis of flow cytometry data revealed that the proportion of CD11b^+^CD11c^high/low^ Mφ/cDCs in the livers (Fig. 2A, first row) of RAG-2^−/−^ RB^high^ and IL-10^−/−^ mice was significantly increased when compared with WT mice. Almost all CD11b^+^CD11c^high/low^ Mφ/cDCs expressed F4/80 (Fig. 2A, third and fourth rows), therefore we classified them as mononuclear phagocyte system cells. In contrast, the proportion of CD11b^−^CD11c^low^PDCA-1^+^ pDCs in the livers of WT mice was significantly higher than those in RAG-2^−/−^ RB^high^ and IL-10^−/−^ mice (Fig. 2A, second row). Statistical analysis confirmed reciprocal changes, where a decrease in the proportion (and absolute number) of pDCs corresponded to an increase in Mφ/cDCs in the liver of colitic mice (Fig. 2B). F4/80^+^ CD11b^+^CD11c^high^ cells, but not F4/80^+^CD11b^+^CD11c^low^ cells, were predominant in hepatic Mφ/cDCs (Fig. 2C). Only a small number of pDCs were found in the LP of the colon under both healthy and colitic conditions (Fig. 2D and E).

### Hepatic Mφ/cDCs Under Colitic Conditions Induce a Th1 Inflammatory Response

**Figure 3:**
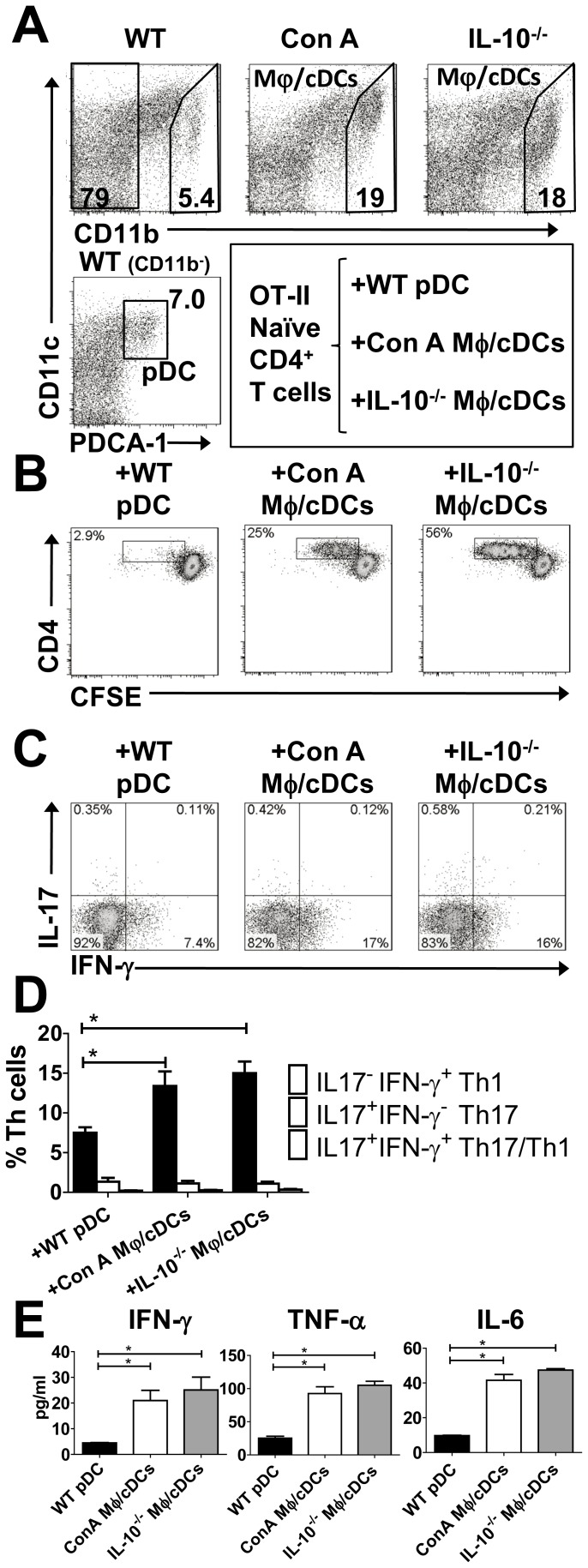
Hepatic Mφ/cDCs cells under colitic conditions induce a Th1 inflammatory response. (**A**) FACS analysis of PDCA-1^+^CD11b^−^CD11c^int^ pDCs from the livers of WT (left column) mice. We also analyzed CD11b^+^CD11c^−^ Mφs from the livers of ConA-treated (middle) and IL-10^−/−^ (right) mice, respectively. Dead cells were excluded with 7AAD staining. (**B**) Proliferation of naïve CFSE-labeled splenic CD4^+^ T cells from OT-II mice, and co-cultured WT pDCs, ConA Mφs, or IL-10^−/−^ Mφs in the presence of OVA. Dead cells were excluded with 7AAD staining and CD4^+^ T cells gated on CD3^+^ CD4^+^ cells are shown (B and C). Data are representative of three independent experiments. (**C**) Intracellular IFN-γ and IL-17A expression in CD4^+^ T cells co-cultured with WT pDCs, ConA Mφs, or IL-10^−/−^ Mφs in the presence of OVA. Data are representative of three independent experiments. (**D**) Proportion of IFN-γ^+^IL-17A^−^, IFN-γ^−^IL-17A^+^, and IFN-γ^+^IL-17A^+^ cells among the Th cell population. (**E**) Cytokine concentrations in the culture supernatant of OT-II CD4^+^ T cells that were co-cultured with WT pDCs or ConA Mφs. Data are representative of three independent experiments. Each experiment was performed using duplicate samples. *P<0.05.

Owing to finding drastic compositional changes of liver APCs in colitic conditions, we assessed the function of hepatic CD11b^−^CD11c^low^PDCA-1^+^ pDCs isolated from the livers of WT mice (WT pDCs), and CD11b^+^CD11c^−/+^ Mφ/cDCs isolated from the livers of colitic IL-10^−/−^ mice (IL-10^−/−^ Mφ/cDCs) (Fig. 3A). The positive controls were Mφ/cDCs isolated from ConA-treated livers (ConA Mφ/cDCs) (Fig. 3A). We co-cultured pDCs or Mφ/cDCs with naïve CFSE-labeled CD4^+^ T cells in the presence of OVA peptides. After 72 h in culture, CD4^+^ T cells had extensively divided in the presence of Mφ/cDCs from not only Con A-treated mice but also colitic IL-10^−/−^ mice, but divided little in the presence of WT pDCs (Fig. 3B). To further assess pro-inflammatory responses of Mφ/cDCs, we examined cytokine production from cultured CD4^+^ T cells. Flow cytometry showed a significant increase in the proportion of IFN-γ-expressing CD4^+^ T cells following co-culture with IL-10^−/−^ Mφ/cDCs. A similar result was seen with ConA Mφ/cDCs; however, there was no significant increase in IL-17-producing CD4^+^ T cells (Fig. 3C and D). Consistent with these data, culture supernatants from CD4^+^ T cells following co-culture with IL-10^−/−^ Mφ/cDCs or ConA Mφ/cDCs exhibited a significant increase in IFN-γ and other pro-inflammatory cytokines such as IL-6 and TNF-α (Fig. 3E).

### Immune Dysregulation in the Liver

**Figure 4:**
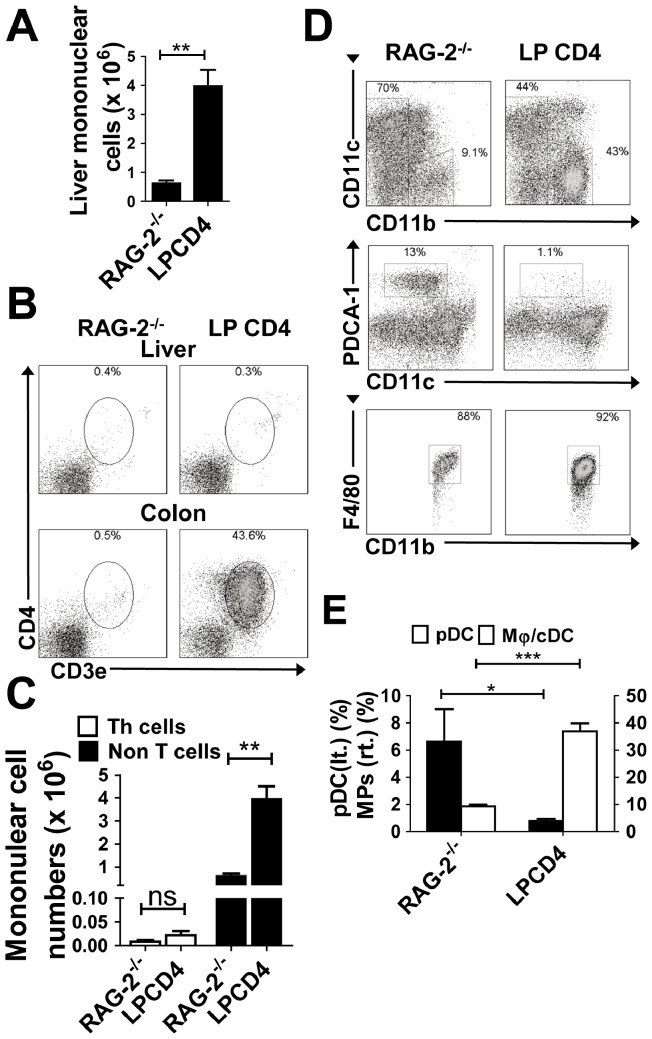
Immune dysregulation in the liver independent of T cell accumulation in the liver. (**A**) Numbers of hepatic mononuclear cells. Data are presented as the mean ± SEM for each group. RAG-2^−/−^ mice (*n* = 4) and RAG-2^−/−^ LP CD4^+^ mice (*n* = 4). (**B**) Representative data from flow cytometry analysis of Th cells in each organ. Dead cells were excluded by 7AAD staining. (**C**) Numbers of hepatic CD3^+^ CD4^+^ Th cells and non-T cells. (**D**) Representative data from flow cytometry analysis of pDCs and Mφs in the liver of each experimental group. Dead cells were excluded using 7AAD staining. Scatter plots for CD11b^−^CD11c^int^ and CD11b^+^CD11c^−^ cells are shown in the middle and bottom rows, respectively. (**E**) Proportion of PDCA-1^+^CD11b^−^CD11c^int^ pDCs and F4/80^+^CD11b^+^CD11c^−^ Mφs among whole mononuclear cells. Data are representative of three independent experiments. Values are presented as the mean ± SEM from seven mice in each group. *P<0.05, **P<0.01, ***P<0.005.

Given the evidence that activated Mφ/cDCs in the liver instruct naïve CD4^+^ T cells to differentiate into Th1 cells (Fig. 3C), we then examined whether the primary recruitment of colitogenic Th1 cells to the liver or other mechanisms induced a dysregulation in the balance of Mφ/cDCs and pDCs in the liver under colitic conditions, as mononuclear cells expanded in the liver in colitic RAG-2^−/−^ RB^high^ mice and IL-10^−/−^ mice (Fig. 1). As an alternative mechanism, the breakdown of the colonic barrier and sequential uptake of MAMPs or other gut-derived antigens during the colitis state may play an important role in drastic changes of APCs in the liver. To minimize the effects of liver-infiltrating T cells, we used an adoptive retransfer system: colitogenic LP CD4^+^ T cells obtained from established RAG-2^−/−^ RB^high^ mice were transferred into RAG-2^−/−^ mice to generate RAG-2^−/−^ LP CD4^+^ mice, as colitogenic CD4^+^ T cells residing in the intestine express gut-specific homing receptors and have an ability to preferentially migrate to the intestine but not to liver [24], [25]. These mice developed severe colitis (data not shown) and also showed significant increases in the number of liver-infiltrating mononuclear cells (Fig. 4A). The RAG-2^−/−^ LP CD4^+^ mice showed almost no CD3^+^CD4^+^ T cell infiltration in the liver, but did exhibit severe colitis with marked infiltrations of CD3^+^CD4^+^ T cells in the colon (Fig. 4B). We confirmed statistically that the significant increases in liver mononuclear cells in RAG-2^−/−^ LP CD4^+^ mice was due to the emergence of non-T cells (possibly APCs) (Fig. 4C). We further investigated compartments of APCs in the liver of RAG-2^−/−^ LP CD4^+^ mice. Consistent with the data from colitic RAG-2^−/−^ RB^high^ or IL-10^−/−^ mice (Fig. 2), RAG-2^−/−^ LP CD4^+^ mice also showed reciprocal changes; a significant decrease in pDCs corresponded with an increase in Mφ/cDCs (Fig. 4D and E). RAG-2^−/−^ LP CD4^+^ mice exhibited severe colitis without infiltration of T cells in the liver (Fig. 4B and 4C). These data suggest that intestinal inflammation induce changes in the compartments of APCs.

### Accumulation of Mφ/cDCs in the Livers of Mice with DSS-induced Colitis

**Figure 5:**
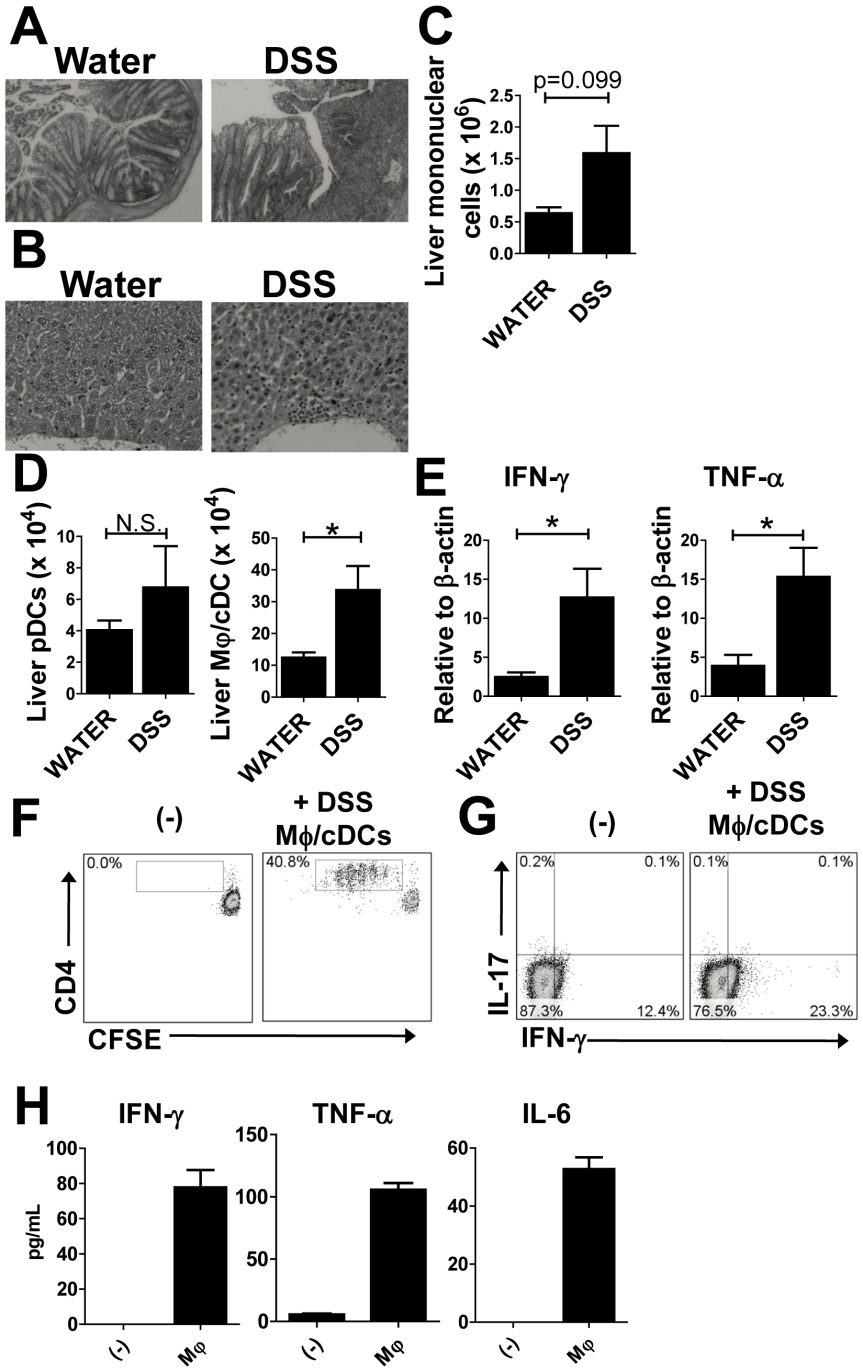
Accumulation of liver macrophages in acute colitis models. (**A**) H&E specimens of the colon taken from mice treated with water (left) or 2% DSS (right). Magnification, ×40 (**B**) H&E specimens of livers from mice treated with water (left) and DSS (right). Magnification, ×100 (**C**) The number of liver mononuclear cells from water- and DSS-treated mice. (**D**) The absolute number of PDCA-1^+^CD11b^−^CD11c^int^ pDCs and CD11b^+^CD11c^−^ Mφs among whole mononuclear cells. Data are representative of three independent experiments. (**E**) Levels of mRNA transcripts for IFN-γ, TNF, and IL-6 in the liver. Values are presented as the mean ± SEM for each group (*n* = 4, water-treated group; *n* = 5, DSS-treated group). *P<0.05. N.S., no significant difference. (**F–H**) Hepatic DSS Mφs induce a Th1 inflammatory response. (**F**) Proliferation of naïve CFSE-labeled splenic CD4^+^ T cells. (**G**) Intracellular IFN-γ and IL-17A expression in naïve CFSE-unlabeled splenic CD4^+^ T cells from OT-II mice that were co-cultured with or without hepatic Mφs from DSS-treated WT mice in the presence of OVA. Dead cells were excluded with 7AAD staining, and CD4^+^ T cells gated on CD3^+^ CD4^+^ cells are shown. Data are representative of two independent experiments. (**H**) Representative cytokine concentrations in culture supernatants from two independent experiments. Each experiment was performed using duplicate samples.

To further determine whether hepatic immune dysregulation is caused by barrier disruption of the intestinal wall, we looked at livers from immune-sufficient WT mice subjected to DSS-induced colitis under SPF conditions. Seven days after the start of DSS administration, mice exhibited severe colitis and infiltration of mononuclear cells in the liver (Fig. 5A and B). Consistent with histological data, liver mononuclear cells were upregulated in DSS-treated mice when compared with water-treated mice (Fig. 5C). Flow cytometry revealed that the number of Mφ/cDCs was significantly increased in the livers of DSS-treated mice; however, there were no significant changes in the numbers of pDCs (Fig. 5D). Expression levels of IFN-γ and TNF-α in the liver were significantly increased in DSS-treated mice (Fig. 5E). Furthermore, hepatic Mφ/cDCs in the DSS-treated mice promoted proliferation of CD4^+^ T cells (Fig. 5F), and increased the proportion of IFN-γ-expressing CD4^+^ T cells (Fig. 5G). We detected a significant increase in pro-inflammtory cytokines in co-culture supernatants (Fig. 5H).

**Figure 6:**
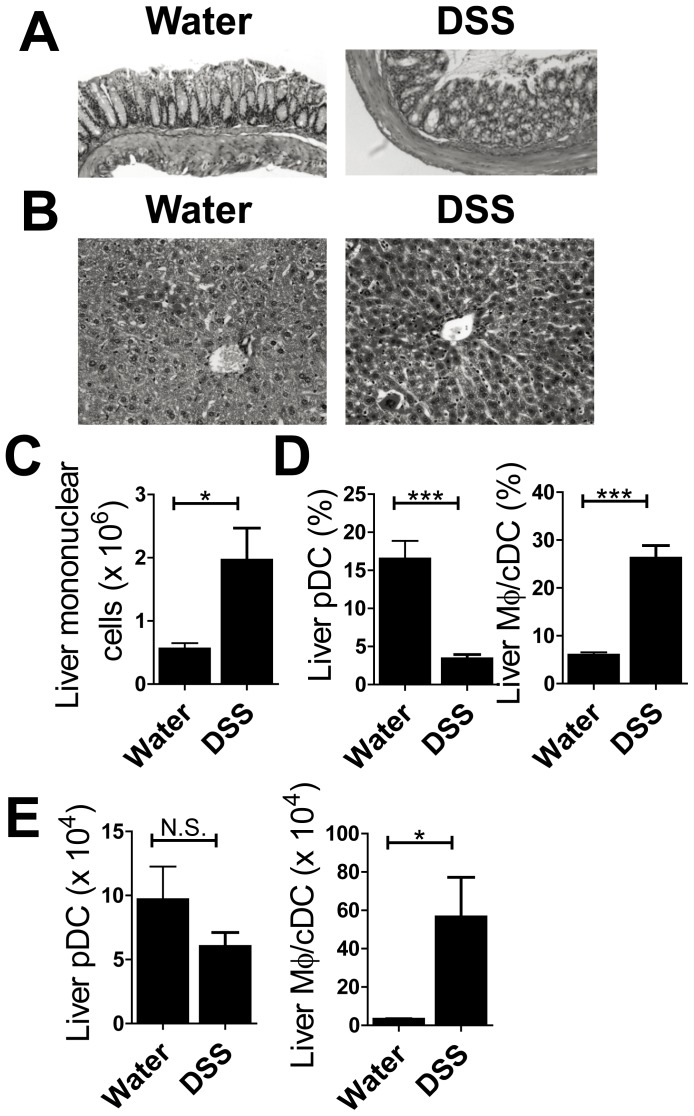
Hepatic infiltration of Macrophages are obserbed in DSS-treated RAG-2^−/−^ mice. (**A**) H&E specimens of colon from mice treated with water (left) or 4% DSS (right). Magnification, ×100 (**B**) H&E specimens of liver from water- (left) and DSS-treated (right) mice. Magnification, ×200 (**C**) The number of liver mononuclear cells for each group of mice. (**D**) Proportion and (**E**) absolute number of PDCA-1^+^CD11b^−^CD11c^int^ pDCs and CD11b^+^CD11c^−^ macrophages among whole mononuclear cells. Data are representative of two independent experiments. Values are presented as the mean ± SEM for each group (*n* = 4, water-treated group; *n* = 4, DSS-treated group). *P<0.05. N.S., no significant difference.

We also confirmed the reciprocal changes for pDCs and Mφ/cDCs in DSS-treated RAG-2^−/−^ mice, which had severe colitis and infiltration of mononuclear cells in the liver (Fig. 6A, B and C). The proportion of pDCs was decreased in DSS-treated RAG-2^−/−^ mice (Fig. 6D). Furthermore, the proportion and number of Mφ/cDCs was increased, even in mice lacking CD4^+^ T cells (Fig. 6D and E). These data suggest that colon inflammation induces the recruitment of Mφ/cDCs and these newly recruited Mφ/cDCs stimulate Th1 cells or promote differentiation of Th1 cells.

**Figure 7:**
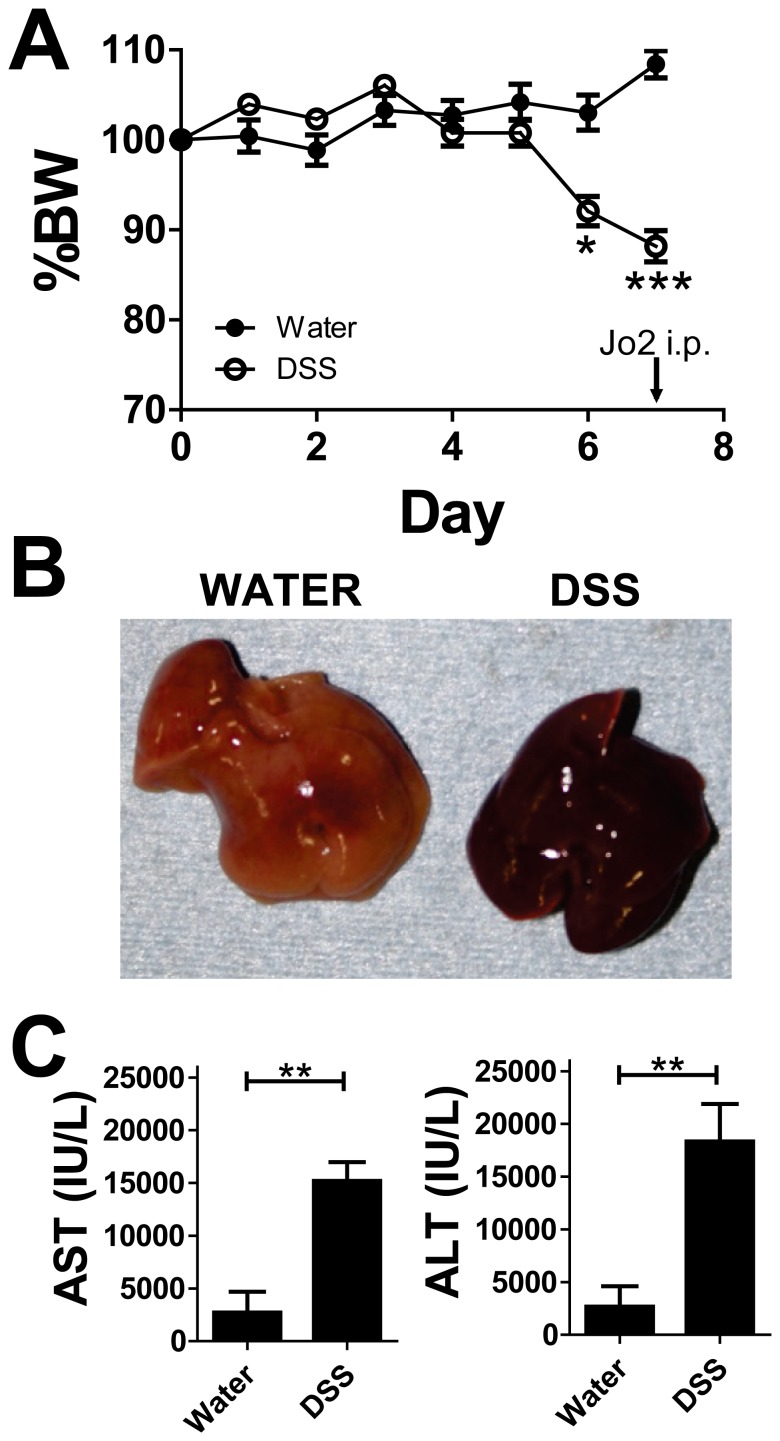
Newly recruited macrophages in the liver during colitis predispose it to inflammation. WT mice were treated under SPF conditions with 2% DSS for 5 days and subsequently with water for 2 days (*n* = 5 mice per group). The Fas-activating antibody, Jo2, was injected i.p. into mice. (**A**) Changes in body weight are expressed as a percentage of original weight. Values are presented as the mean ± SEM for each group. Data are representative of two independent experiments. (**B**) Macroscopic view of livers from water- (left) and DSS-treated mice. (**C**) Levels of aspartate aminotransferase (left) and alanine aminotransferase (right) in water- and DSS-treated mice 6 h after Jo2 injection.

We also assessed whether these Mφ/cDCs that emerged under colitic conditions contributed to acute liver inflammation, which had been initially induced by Fas-activating antibody (Jo2) [18], [19]. As shown in Fig. 7A, DSS-treated mice with significant body weight loss underwent Jo2 treatment. The livers of DSS-induced colitic mice in which Jo2 was administered showed significant blood accumulation (Fig. 7B). Consistently, the levels of aspartate aminotransferase and alanine aminotransferase were significantly increased in DSS mice after Jo2 treatment compared with non-DSS mice (Fig. 7C).

### Leukocyte Infiltration was not Detected in Acute Colitis Models Under GF Conditions

**Figure 8:**
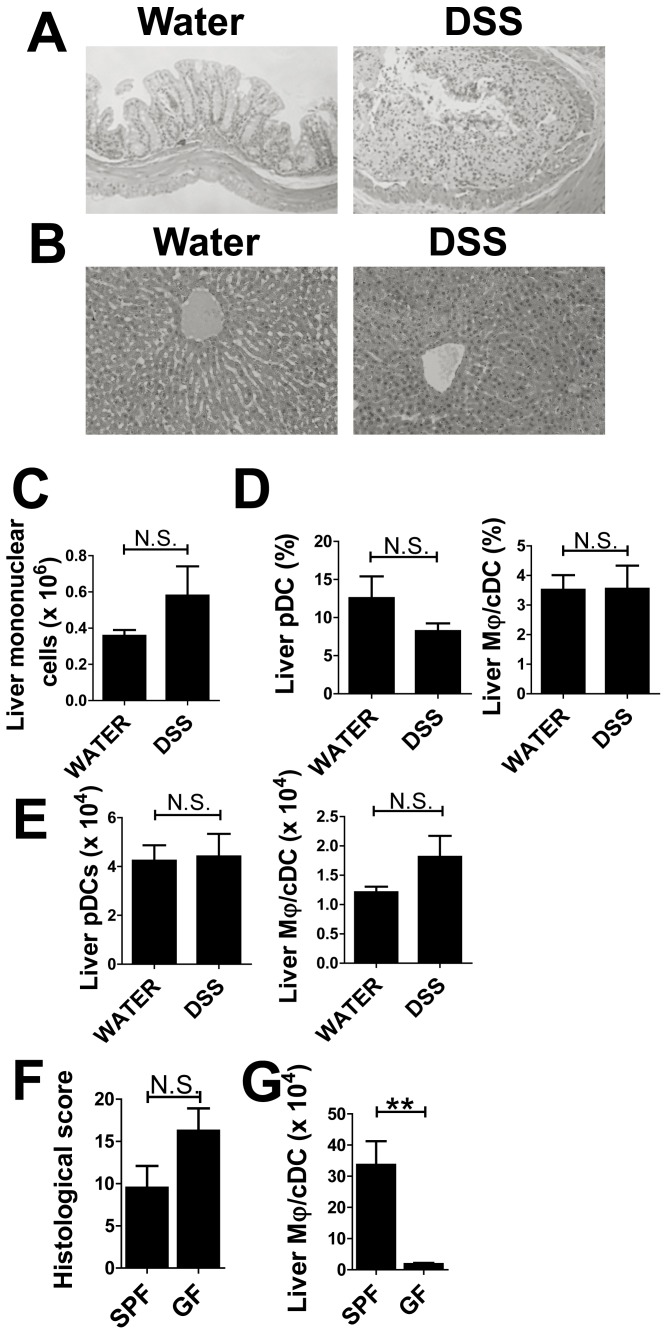
GF condition abrogates the compositional changes of hepatic APCs in acute colitis models. (**A**) H&E staining of colon sections taken from mice treated with water (left) or DSS (right). Magnification, ×100. (**B**) H&E staining of liver sections from water- (left) and DSS-treated (right) mice. Magnification, ×100. (**C**) Number of liver mononuclear cells. (**D**) Proportion and (**E**) absolute number of PDCA-1^+^CD11b^−^CD11c^int^ pDCs and CD11b^+^CD11c^−^ Mφs among whole mononuclear cells. (**F, G**) Comparisons between SPF and GF in the histology (F) and the numbers of Mφs (G) in DSS-treated mice. Data are representative of two independent experiments. Values are presented as the mean ± SEM for each group (*n* = 5, water-treated GF group; *n* = 4, DSS-treated GF group; *n* = 5, DSS-treated SPF group). N.S., no significant difference.

We investigated whether MAMPs or other bacterial degradation products induce hepatic immune dysregulation and analyzed the livers of mice treated with DSS under GF conditions. Bacteria do not reside in the intestines of GF mice, meaning there is no inflow of bacterial components into the liver. Mice treated with DSS under GF conditions showed severe colitis; however, there was no evidence of leukocyte infiltration (Fig. 8A and B). Consistent with histological data, we observed no significant changes in the number of liver mononuclear cells for controls and DSS-treated mice (Fig. 8C). Flow cytometry data showed that there were no significant changes in the ratios of pDCs to Mφ/cDCs (Fig. 8D); or in the absolute numbers of these cells in the livers of DSS-treated mice (Fig. 8E). DSS-treated GF mice also exhibited severe colitis compared with SPF mice (Fig. 8F), but Mφ/cDCs were not increased (Fig. 8G). These findings indicate that bacterial products play a crucial role in inducing infiltration of Mφ/cDCs into the liver.

## Discussion

In this study we demonstrated: (1) hepatic pDCs are decreased and Mφ/cDCs are increased in mice with chronic intestinal inflammation; (2) newly emerged Mφ/cDCs during the development of colitis possess pro-inflammatory characteristics that drive differentiation of naïve T cells toward Th1 cells; (3) Mφ/cDCs that emerge during colitis possibly result in the exacerbation of hepatitis symptoms; and (4) the reciprocal changes we observed in the compartments of the liver’s innate immune system during intestinal inflammation were mainly caused by MAMPs, other bacterial degradation products, or bacteria themselves subsequent to the disruption of the intestinal wall. Changes in APC compartments were seen not only in RAG-2^−/−^ RB^high^ mice and DSS-administered mice but also in RAG-2^−/−^ mice retransferred with gut-tropic colitogenic LP CD4^+^ T cells in SPF conditions; but were not seen in mice of the DSS colitis model in the GF condition that lack commensal bacteria in the gut.

Previous studies have suggested a relationship between intestinal and liver inflammation [2], [3], [11], [26]. These previous reports support our hypothesis that intestinal inflammation skews the balance of immune cells in the liver. However, no expansive research has been conducted to clarify the inflammatory relationship between the liver and the intestine. In this study, we are the first to demonstrate distinctive changes in compartments (pDCs vs. Mφ/cDCs) of hepatic immune cells due to chronic intestinal inflammation. Increased hepatic Mφ/cDCs appeared irrespective of whether the colitis model was acute or chronic. Immunological changes were not only observed in the liver during colitis, but also in ConA-induced liver injury (Fig. 3A) [14], suggesting that these changes are universal phenomena during liver stress.

We hoped to elucidate how these crucial changes of hepatic APCs occur during intestinal inflammation. The liver is between the portal and systemic circulatory systems. The liver receives continuous blood supplements from the intestine via the portal vein and is presumably exposed to MAMPs or other degradation products from viable or non-viable commensal bacteria [8]. We used a GF system to demonstrate the importance of bacterial components in causing immunological dysregulation in the liver. Most models of experimental colitis fail to develop under GF conditions [27], [28]; however, we used a DSS model, which is known to result in the development of severe colitis [29], [30]. DSS-treated mice under SPF conditions exhibited Mφ/cDC infiltration into the liver (Fig. 5 and 8). This was not observed for DSS-treated mice under GF conditions despite the existence of severe colitis. This means that the accumulation of Mφ/cDCs is not just a consequence of nonspecific inflammation related with colitis. Mice under GF conditions lack the protective effects against colitis from microbiota [31], but also lack the stimulant transferred from the intestine to the liver. These data suggest that stimulation from degradation products from intestinal commensal bacteria play an important role in recruitment of Mφ/cDCs.

Various commensal bacteria that usually reside in the intestine (such as *C. coccoides*, *C. leptum* and *Enterococcus)* were found in ConA-treated and untreated livers [20]. Such a finding lends support to the hypothesis that intestinal bacterial products or bacteria themselves are transferred from the intestine to the liver. The rate (and amount) of uptake of bacteria-derived products, such as microbial DNA and LPS, is thought to increase during colitis because of the fragility of the colon wall [11], [32]. Further study is required to estimate the amount and type of bacterial products that stimulate the liver during colitis.

Despite our finding that recruitment of Mφ/cDCs is stimulated by bacterial degradation products or bacteria themselves, several scenarios may be considered as additional mechanisms underlying the accumulation of Mφ/cDCs. First, activated Mφ/cDCs themselves migrate from the intestine to the liver. Alternatively, circulating monocytes accumulate in the liver stimulated by pro-inflammatory cytokines transferred via the portal vein, such as TNF-α produced by LP CD4^+^ T cells or APCs in the intestine [30]. However, pro-inflammatory cytokines are produced in the colon of GF mice, so this possibility may only have a partial effect. Whether increased Mφ/cDCs originate from monocytes or resident macrophages in the liver should be explored in future studies. Third, activated T cells migrate from the intestine to the liver and stimulate the liver to recruit and activate circulating or resident Mφ/cDCs. RAG-2^−/−^ LP CD4^+^ mice (Fig. 3) and DSS-treated RAG-2^−/−^ (Fig. 6) mice show increased Mφ/cDCs without infiltration of T, B, and NKT cells in the liver, which suggests that Mφ/cDCs are recruited to the liver independently of T, B, and NKT cells. However, there still remains involvement of cytokines, DSS itself and other types of the cells such as NK cells and liver sinusoidal endothelial. Macrophages accumulated during both acute and chronic colitis models in the liver produced inflammatory cytokines and promoted differentiation of Th1 cells or activation of NK cells. Thus, it is likely that systemic IFN-γ production leads subsequent upregulation of the sensitivity of FAS-mediated signal in the liver.

The current study suggests that hepatic APC compartments alter in parallel with the progression of colitis, and increased Mφ/cDCs have pro-inflammatory characteristics. Two major hepatic diseases presenting as extraintestinal manifestations in IBD patients are PSC and autoimmune hepatitis. The prevalence of these liver diseases is reported, both in Crohn’s disease and ulcerative colitis, to correlate with the severity and expansion of the intestinal disease [3], [26], [33]. The active disease is associated with ongoing extraintestinal manifestations in patients with Crohn’s disease [26]. The prevalence of PSC was 5.5% in patients with substantial colitis and 0.5% in patients with distal colitis [2]. Therefore, Mφ/cDCs infiltrating into the liver in the colitis models may be involved in the pathogenesis of IBD-related liver diseases. However, liver enzymes demonstrated no significant changes during colitis unlike autoimmune hepatitis or PSC. Thus, we hypothesized that accumulated Mφ/cDCs may increase the susceptibility of hepatitis. We combined the Fas-mediated model of hepatitis with the DSS colitis models to show the clinical importance of our study. Fas-mediated hepatitis models are widely used as a model of hepatitis [18], [19]. TNF-α released by activated hepatic macrophages is one of the very important factors that damage hepatocytes, which are highly sensitive to cell-extrinsic stimulation in Fas-mediated hepatitis [19]. We also suggest the importance of the infiltration of macrophages in fulminant hepatitis models [10]. Mφ/cDCs recruited to the liver during colitis in a T cell-independent manner produce pro-inflammatory cytokines and promote Th1 reaction. CD11b^+^CD11c^low/+^ Mφ/cDCs are already detectable in healthy WT mice (Fig. 2B), but previous studies suggest that these resident macrophages including Kupffer cells have a immunoregulatory character, such as producing IL-10 [6], [34]. Taken together with the previous studies and our findings, Mφ/cDCs infiltrating into the liver during colitis may contribute to making the hepatitis worse. Some additional stimulation would be needed for breaking down liver tolerance and causing hepatitis or cholangitis which mimics liver diseases associated with IBD.

An additional finding of our study is that pDCs are significantly decreased in the late phase of colitis in the chronic models. Hepatic pDCs plays an important role in maintaining liver homeostasis [9], [10], [14], so not only increased Mφ/cDCs but decreased pDCs may have effects on the infiltration of mononuclear cells in the liver during chronic colitis. This is known as sustained negative regulation of LPS signaling in response to a second LPS stimulus. pDCs express certain toll-like receptors (TLR) [14], with the number of spleen pDCs decreasing following TLR treatment [35]. These previous reports suggest that pDCs are one of the initial sensors of MAMPs or other degradation products, and possibly decrease when the endotoxin tolerance is broken down. Further studies will be needed to address this issue.

In conclusion, the findings from our study suggest that dysregulation in the balance of liver APCs, especially in the recruitment of pro-inflammatory Mφ/cDCs, during colitis occurs in a T-cell-independent manner for chronic and acute murine colitis models. This report may provide the basis for a novel strategy to treat intractable immune diseases in the liver. We have also highlighted the relevance of the relationship between liver and intestine immunology during intestinal inflammation.

## References

[1] Baumgart DC, Sandborn WJ (2007) Inflammatory bowel disease: clinical aspects and established and evolving therapies. Lancet 369: 1641–1657. 17499606 10.1016/S0140-6736(07)60751-X

[2] Olsson R, Danielsson A, Jarnerot G, Lindstrom E, Loof L, et al (1991) Prevalence of primary sclerosing cholangitis in patients with ulcerative colitis. Gastroenterology 100: 1319–1323. 2013375

[3] Rasmussen HH, Fallingborg JF, Mortensen PB, Vyberg M, Tage-Jensen U, et al (1997) Hepatobiliary dysfunction and primary sclerosing cholangitis in patients with Crohn’s disease. Scand J Gastroenterol 32: 604–610. 9200295 10.3109/00365529709025107

[4] Tremaroli V, Backhed F (2012) Functional interactions between the gut microbiota and host metabolism. Nature 489: 242–249. 22972297 10.1038/nature11552

[5] Kaisho T, Takeuchi O, Kawai T, Hoshino K, Akira S (2001) Endotoxin-induced maturation of MyD88-deficient dendritic cells. J Immunol 166: 5688–5694. 11313410 10.4049/jimmunol.166.9.5688

[6] Crispe IN (2009) The liver as a lymphoid organ. Annu Rev Immunol 27: 147–163. 19302037 10.1146/annurev.immunol.021908.132629

[7] Crispe IN (2003) Hepatic T cells and liver tolerance. Nat Rev Immunol 3: 51–62. 12511875 10.1038/nri981

[8] Thomson AW, Knolle PA (2010) Antigen-presenting cell function in the tolerogenic liver environment. Nat Rev Immunol 10: 753–766. 20972472 10.1038/nri2858

[9] Goubier A, Dubois B, Gheit H, Joubert G, Villard-Truc F, et al (2008) Plasmacytoid dendritic cells mediate oral tolerance. Immunity 29: 464–475. 18789731 10.1016/j.immuni.2008.06.017 PMC3545652

[10] Tokita D, Sumpter TL, Raimondi G, Zahorchak AF, Wang Z, et al (2008) Poor allostimulatory function of liver plasmacytoid DC is associated with pro-apoptotic activity, dependent on regulatory T cells. J Hepatol 49: 1008–1018. 18926588 10.1016/j.jhep.2008.07.028 PMC2631180

[11] Gabele E, Dostert K, Hofmann C, Wiest R, Scholmerich J, et al (2011) DSS induced colitis increases portal LPS levels and enhances hepatic inflammation and fibrogenesis in experimental NASH. J Hepatol 55: 1391–1399. 21703208 10.1016/j.jhep.2011.02.035

[12] Kamada N, Hisamatsu T, Okamoto S, Sato T, Matsuoka K, et al (2005) Abnormally differentiated subsets of intestinal macrophage play a key role in Th1-dominant chronic colitis through excess production of IL-12 and IL-23 in response to bacteria. J Immunol 175: 6900–6908. 16272349 10.4049/jimmunol.175.10.6900

[13] Kamada N, Hisamatsu T, Okamoto S, Chinen H, Kobayashi T, et al (2008) Unique CD14 intestinal macrophages contribute to the pathogenesis of Crohn disease via IL-23/IFN-gamma axis. J Clin Invest 118: 2269–2280. 18497880 10.1172/JCI34610 PMC2391067

[14] Nakamoto N, Ebinuma H, Kanai T, Chu PS, Ono Y, et al (2012) CCR9(+) Macrophages Are Required for Acute Liver Inflammation in Mouse Models of Hepatitis. Gastroenterology 142: 366–376. 22079594 10.1053/j.gastro.2011.10.039

[15] Powrie F, Leach MW, Mauze S, Caddle LB, Coffman RL (1993) Phenotypically distinct subsets of CD4+ T cells induce or protect from chronic intestinal inflammation in C. B-17 scid mice. Int Immunol 5: 1461–1471. 7903159 10.1093/intimm/5.11.1461

[16] Ishii K, Kanai T, Totsuka T, Uraushihara K, Ishikura T, et al (2004) Hyperexpression of inducible costimulator on lamina propria mononuclear cells in rat dextran sulfate sodium colitis. J Gastroenterol Hepatol 19: 174–181. 14731127 10.1111/j.1440-1746.2004.03202.x

[17] Kuhn R, Lohler J, Rennick D, Rajewsky K, Muller W (1993) Interleukin-10-deficient mice develop chronic enterocolitis. Cell 75: 263–274. 8402911 10.1016/0092-8674(93)80068-p

[18] Murthy A, Defamie V, Smookler DS, Di Grappa MA, Horiuchi K, et al (2010) Ectodomain shedding of EGFR ligands and TNFR1 dictates hepatocyte apoptosis during fulminant hepatitis in mice. J Clin Invest 120: 2731–2744. 20628198 10.1172/JCI42686 PMC2913323

[19] Ogasawara J, Watanabe-Fukunaga R, Adachi M, Matsuzawa A, Kasugai T, et al (1993) Lethal effect of the anti-Fas antibody in mice. Nature 364: 806–809. 7689176 10.1038/364806a0

[20] Ojiro K, Ebinuma H, Nakamoto N, Wakabayashi K, Mikami Y, et al (2010) MyD88-dependent pathway accelerates the liver damage of Concanavalin A-induced hepatitis. Biochem Biophys Res Commun 399: 744–749. 20696131 10.1016/j.bbrc.2010.08.012

[21] Mikami Y, Kanai T, Sujino T, Ono Y, Hayashi A, et al (2010) Competition between colitogenic Th1 and Th17 cells contributes to the amelioration of colitis. Eur J Immunol 40: 2409–2422. 20706984 10.1002/eji.201040379

[22] Kimura K, Kanai T, Hayashi A, Mikami Y, Sujino T, et al (2012) Dysregulated balance of retinoid-related orphan receptor gammat-dependent innate lymphoid cells is involved in the pathogenesis of chronic DSS-induced colitis. Biochem Biophys Res Commun 427: 694–700. 23022186 10.1016/j.bbrc.2012.09.091

[23] Geissmann F, Manz MG, Jung S, Sieweke MH, Merad M, et al (2010) Development of monocytes, macrophages, and dendritic cells. Science 327: 656–661. 20133564 10.1126/science.1178331 PMC2887389

[24] Mora JR, Bono MR, Manjunath N, Weninger W, Cavanagh LL, et al (2003) Selective imprinting of gut-homing T cells by Peyer’s patch dendritic cells. Nature 424: 88–93. 12840763 10.1038/nature01726

[25] Nemoto Y, Kanai T, Kameyama K, Shinohara T, Sakamoto N, et al (2009) Long-lived colitogenic CD4+ memory T cells residing outside the intestine participate in the perpetuation of chronic colitis. J Immunol 183: 5059–5068. 19786550 10.4049/jimmunol.0803684

[26] Vavricka SR, Brun L, Ballabeni P, Pittet V, Prinz Vavricka BM, et al (2011) Frequency and risk factors for extraintestinal manifestations in the Swiss inflammatory bowel disease cohort. Am J Gastroenterol 106: 110–119. 20808297 10.1038/ajg.2010.343

[27] Podolsky DK (2002) Inflammatory bowel disease. N Engl J Med 347: 417–429. 12167685 10.1056/NEJMra020831

[28] Sartor RB (2006) Mechanisms of disease: pathogenesis of Crohn’s disease and ulcerative colitis. Nat Clin Pract Gastroenterol Hepatol 3: 390–407. 16819502 10.1038/ncpgasthep0528

[29] Hayashi A, Sato T, Kamada N, Mikami Y, Matsuoka K, et al (2013) A Single Strain of Clostridium butyricum Induces Intestinal IL-10-Producing Macrophages to Suppress Acute Experimental Colitis in Mice. Cell Host Microbe 13: 711–722. 23768495 10.1016/j.chom.2013.05.013

[30] Maslowski KM, Vieira AT, Ng A, Kranich J, Sierro F, et al (2009) Regulation of inflammatory responses by gut microbiota and chemoattractant receptor GPR43. Nature 461: 1282–1286. 19865172 10.1038/nature08530 PMC3256734

[31] Rakoff-Nahoum S, Paglino J, Eslami-Varzaneh F, Edberg S, Medzhitov R (2004) Recognition of commensal microflora by toll-like receptors is required for intestinal homeostasis. Cell 118: 229–241. 15260992 10.1016/j.cell.2004.07.002

[32] Henao-Mejia J, Elinav E, Jin C, Hao L, Mehal WZ, et al (2012) Inflammasome-mediated dysbiosis regulates progression of NAFLD and obesity. Nature 482: 179–185. 22297845 10.1038/nature10809 PMC3276682

[33] Bjornsson E, Olsson R, Bergquist A, Lindgren S, Braden B, et al (2008) The natural history of small-duct primary sclerosing cholangitis. Gastroenterology 134: 975–980. 18395078 10.1053/j.gastro.2008.01.042

[34] Knolle P, Schlaak J, Uhrig A, Kempf P, Meyer zum Buschenfelde KH, et al (1995) Human Kupffer cells secrete IL-10 in response to lipopolysaccharide (LPS) challenge. J Hepatol 22: 226–229. 7790711 10.1016/0168-8278(95)80433-1

[35] Swiecki M, Wang Y, Vermi W, Gilfillan S, Schreiber RD, et al (2011) Type I interferon negatively controls plasmacytoid dendritic cell numbers in vivo. J Exp Med 208: 2367–2374. 22084408 10.1084/jem.20110654 PMC3256963

